# Effects of Prediabetes on Ventricular Repolarization Markers in Electrocardiography

**DOI:** 10.31083/RCM26266

**Published:** 2025-02-19

**Authors:** Tolga Memioğlu, Mehmet İnanır, Kenan Toprak, Müjgan Gürler

**Affiliations:** ^1^Faculty of Medicine, Bolu Abant Izzet Baysal University, 14030 Bolu, Turkey; ^2^Faculty of Medicine, Harran University, 63050 Sanliurfa, Turkey

**Keywords:** arrhythmia, electrocardiography, prediabetes, repolarization marker, Tp-e interval

## Abstract

**Background:**

The blood glucose levels in people with prediabetes mellitus (PDM) are regarded as too high to be normal but below the cutoff for diabetes mellitus (DM). Clinical indicators for PDM patients include impaired glucose tolerance (IGT), impaired fasting glucose (IFG), and/or hemoglobin A1c (HbA1c) levels between 5.7 and 6.4% (39–47 mmol/mol). PDM has been shown to raises the risk of cardiovascular disease (CVD) and mortality. Meanwhile, death and morbidity can be predicted by the new ventricular repolarization features of the electrocardiogram (ECG). Several studies have analyzed the connection between DM and the ventricular repolarization characteristics of ECG; however, few studies have examined the connection between PDM and these ventricular repolarization characteristics. This study evaluated the ECG ventricular repolarization parameters in individuals with PDM.

**Methods:**

A retrospective case-control design was used. Randomly selected participants included 79 PDM patients (30 men, mean age: 39.7 ± 5.7 years) and 79 controls (30 men, mean age: 39.8 ± 5.2 years). ECG intervals analyzed were the distance from the beginning of the Q wave to the end of the T wave (QT), the distance between Q and S waves (QRS), the distance between the T wave’s termination and point J (JT), and the distance between the peak and endpoint of the T wave (Tp-e), along with corrected and derived measures (corrected QT interval (QTc), the difference between the maximum and smallest QT intervals (QTd), corrected QTd (QTdc), corrected JT interval (JTc), Tp-e/QT, Tp-e/QTc, Tp-e/JT, Tp-e/JTc). Patient records were retrieved from the institution’s database.

**Results:**

Both groups had comparable averages for age, gender, smoking, hyperlipidemia, body mass index (BMI), (*p* > 0.05 for each). Similarly, both groups had similar QT, QRS, and JT intervals. PDM patients had significantly greater heart rates (bpm), and QTc, QTd, QTdc, JTc, and Tp-e intervals (millisecond, ms) than the control group. The results were deemed significant when HbA1c levels were associated with every employed ECG measurement in our investigation.

**Conclusions:**

In our study, the HbA1c value was shown to be moderately positively correlated with the heart rate and QTc, QTd, QTdc, JTc, and Tp-e intervals, all of which were determined to be significant. Additionally, the HbA1c value showed a weak positive correlation with Tp-e/QT, Tp-e/JT ratios, which were statistically significant. This study showed that patients with PDM are prone to ventricular arrhythmia in the early period of the disorder.

## 1. Introduction

Globally, prediabetes mellitus (PDM) is becoming more common, with stress, urbanization, dietary changes (such as consuming more high-fat and high-glycemic meals), sedentary lifestyles, and obesity common contributors to this increase. PDM is applied to people whose blood glucose levels are higher than normal but not high enough to be classified as diabetes mellitus (DM) [1]. Impaired glucose tolerance (IGT) and impaired fasting glucose (IFG) levels are indicators of PDM [2]. Moreover, the American Diabetes Association (ADA) defines PDM as a hemoglobin A1c (HbA1c) level that falls between 5.7% and 6.4%, a baseline blood glucose level of 100–125 mg/dL, or a 2-hour plasma glucose level of 140–199 mg/dL after a 75 g oral glucose load [3]. A single measurement that meets these criteria is sufficient for a PDM diagnosis. Furthermore, type 2 DM is estimated to develop within five years of an IGT or IFG diagnosis in 26% and 50% of cases, respectively [4].

DM is among the most significant global health issues of the 21st century, with an estimated 541 million people affected by IGT in 2021 [2]. Since PDM is linked to a higher risk of cardiovascular disease, it is advised to screen PDM patients for and treat modifiable cardiovascular risk factors [3]. The HbA1c test is a valuable tool for diagnosing and managing DM. Additionally, DM patients with stable glycemia are recommended to undergo the HbA1c test at least twice annually; meanwhile, patients with unstable glycemia may need testing every three months [5]. PDM and type 2 DM share common risk factors, including those leading to PDM development [6]. Indeed, autonomic neuropathy can lead to arrhythmias of unknown origin in individuals with DM or PDM [7]. However, an electrocardiogram (ECG) can detect this condition since it is linked to malignant ventricular arrhythmias. Notably, these also promote a higher mortality in patients with PDM. The distance between the peak and endpoint of the T wave (Tp-e) interval is a possible marker of total repolarization dispersion. Indices derived from a 12-lead ECG, such as the Tp-e interval, the distance from the beginning of the Q wave to the end of the T wave (QT), corrected QT interval (QTc), the distance between the T wave’s termination and point J (JT), and corrected JT interval (JTc) ratios, have been connected to a higher incidence of ventricular arrhythmias and are suggestive of complete repolarization dispersion [8]. This study aimed to assess ventricular repolarization parameters in individuals with PDM.

## 2. Methods

Sample size calculation: A prior power analysis was conducted to ensure adequate power for detecting a medium effect size (Cohen’s d = 0.50). The analysis calculated a significance level of 0.05 and a desired power of 0.85, indicating a required minimum of 73 participants per group. This calculation was based on two-tailed independent samples *t*-tests and was performed using the software G*Power version 3.1.9.7 (Institute for Experimental Psychology, Düsseldorf, North Rhine-Westphalia, Germany). This retrospective case–control study included 79 PDM patients (30 men, mean age 39.7 ± 5.7 years) and a control group of 79 individuals (30 men, mean age 39.8 ± 5.2 years).

QT, the distance between Q and S waves (QRS), JT in the T wave, and Tp-e intervals in the ECGs of the patients and control groups were measured. The QTc, the difference between the maximum and smallest QT intervals (QTd), corrected QTd (QTdc), and JTc intervals were used to calculate the Tp-e/QT, Tp-e/QTc, Tp-e/JT, and Tp-e/JTc ratios.

The local ethics commission approved the study (2019/288). This study adhered to the ethical requirements of the Declaration of Helsinki on biomedical research involving humans. All medical histories were obtained from the institution’s database, and all ECGs were archived. PDM diagnoses followed the ADA criteria [2], with all participants selected from those diagnosed with PDM. The exclusion criteria included patients diagnosed with DM, those with a history of atherosclerotic cardiovascular disease (CVD) and ventricular arrhythmia, severe degree valvular disease, severe heart, liver, or renal failure, chronic lung disease, severe obstructive sleep apnea, electrolyte imbalances, left-axis deviation, atrial fibrillation, and hypertrophic findings.

**Fig. 1.**
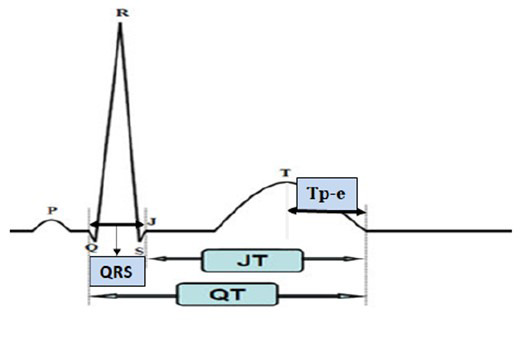
**Electrocardiogram (ECG) arrhythmia indicators**. JT, the distance between the T wave’s termination and point J; QT, the distance from the beginning of the Q wave to the end of the T wave; QRS, the distance between Q and S waves; Tp-e, the distance between the peak and endpoint of the T wave. This figure is quoted from [9].

The ECG was performed with the patient in a supine position using the Nihon Kohden Cardiofax 12-lead ECG-1950 VET equipment (Shinjuku, Tokyo, Japan) at a speed of 25 mm/s and an amplitude of 10 mm/mV. The TorQ 150 mm Digital Caliper LCD gadget was used to manually measure the QRS duration, QT interval, JT interval, and Tp-e interval. Fig. 1 (Ref. [9]) shows the areas of these intervals on the ECG. The Tp-e interval was used to calculate the Tp-e/QT, Tp-e/QTc, Tp-e/JT, and Tp-e/JTc ratios. The Fridericia formula [10] was used to calculate the following additional intervals: QT, QTd, QTdc, QRS, JT, QTc, and JTc. Notably, the Tp-e interval was not corrected for heart rate, as recommended by Andršová *et al*. [11], who noted that the Tp-e interval is not consistently dependent on heart rate.

**Fig. 2.**
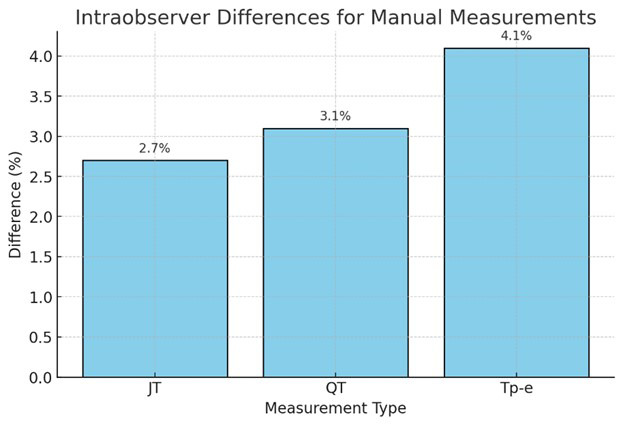
**Intraobserver differences between cardiologists**. JT, the distance between the T wave’s termination and point J; QT, the distance from the beginning of the Q wave to the end of the T wave; Tp-e, the distance between the peak and endpoint of the T wave.

An echocardiography (ECHO) was performed for all patients and calculations of ejection fraction (EF) <50% or indications of severe valve disease were removed from the study. Since patients whose ECHO report was normal were included in the study, no data were recorded separately for procedures. All measurements were conducted manually and double-anonymized by two cardiologists who were unaware of the identities of the patients. There were intraobserver differences between cardiologists in JT, QT, and Tp-e measurements, whose rates were recorded as 2.7%, 3.1%, and 4.1%, respectively. Fig. 2 shows the graph of intraobserver differences among the cardiologists who conducted the study.

### Statistical Analysis

The statistical analysis was conducted using SPSS version 22.0 (IBM Co., Armonk, NY, USA). The Kolmogorov–Smirnov test was employed to assess normality. Quantitative variables are represented by the mean ± standard deviation (SD), whereas categorical data were displayed using the median (min–max value). Mann–Whitney U tests were utilized for non-normally distributed variables; meanwhile, the Student’s *t*-test and Chi-square test were implemented for comparisons. Relationships between PDM, HbA1c, and ventricular repolarization features were examined using Pearson correlation analysis. Statistical significance was defined as a *p*-value of less than 0.05.

## 3. Results

Random selection was used to choose the study and control groups. The PDM group comprised 79 individuals (30 men, mean age 39.7 ± 5.7 years), and the control group included 79 individuals (30 men, mean age 39.8 ± 5.2 years). Both groups were compared regarding age, gender distribution, smoking status, hyperlipidemia, and body mass index (BMI) (*p* > 0.05 for each). HbA1c levels were significantly higher in the PDM group (5.97 ± 0.28 vs 4.06 ± 0.59) (*p* < 0.001 for each). Table 1 displays the demographic differences between the control and PDM groups.

**Table 1.** **The baseline features of the study group**.

	PDM (n = 79)	Control (n = 79)	*p*-value
Age (years)	39.74 ± 5.66	39.84 ± 5.22	0.905
Male/female	30/49	30/49	1.000
Hypertension	5/79	3/79	0.471
Smoking	10/79	10/79	1.000
Hyperlipidemia	4/79	7/79	0.351
BMI (kg/m^2^)	27.21 ± 3.72	26.42 ± 3.92	0.236
HbA1c (%)	5.97 ± 0.28	4.06 ± 0.59	<0.001

PDM, prediabetes mellitus; BMI, body mass index; HbA1c, hemoglobin A1c.

Table 2 shows the ECG findings of the study population. QT (371.7 ± 29.9 vs. 363.58 ± 21.54 ms; *p* = 0.056), QRS (91.46 ± 12.11 vs. 89.01 ± 11.32 ms; *p* = 0.199), JT (284.64 ± 33.39 vs. 280.40 ± 24.19 ms; *p* = 0.372) intervals were similar in both groups. Heart rate (78.6 ± 13.7 vs. 66.66 ± 8.53 bpm; *p* < 0.001) and Tp-e/QT (0.29 ± 0.27 vs. 0.21 ± 0.03; *p* = 0.006), Tp-e/QTc (0.26 ± 0.26 vs. 0.20 ± 0.03; *p* = 0.033), Tp-e/JT (0.40 ± 0.43 vs. 0.27 ± 0.05; *p* = 0.011), Tp-e/JTc (0.35 ± 0.41 vs. 0.26 ± 0.05; *p* = 0.044) ratios were statistically significantly higher in the PDM group. QTc (420.91 ± 25.77 vs. 381.66 ± 23.31; *p* < 0.001), QTd (28.41 ± 8.82 vs. 15.56 ± 6.05; *p* < 0.001), QTdc (32.80 ± 11.37 vs. 16.34 ± 6.60; *p* < 0.001), JTc (322.04 ± 24.47 vs. 294.34 ± 25.07; *p* < 0.001), Tp-e (91.82 ± 11.82 vs. 74.62 ± 10.23; *p* < 0.001) intervals were statistically significantly longer in the PDM group.

**Table 2.** **ECG results for the research participants**.

	PDM (n = 79)	Control (n = 79)	*p*-value
Heart rate (bpm)	78.6 ± 13.7	66.66 ± 8.53	<0.001
QT ms	371.7 ± 29.9	363.58 ± 21.54	0.056
QTc ms	420.91 ± 25.77	381.66 ± 23.31	<0.001
QTd ms	28.41 ± 8.82	15.56 ± 6.05	<0.001
QTdc ms	32.80 ± 11.37	16.34 ± 6.60	<0.001
QRS ms	91.46 ± 12.11	89.01 ± 11.32	0.199
JT ms	284.64 ± 33.39	280.40 ± 24.19	0.372
JTc ms	322.04 ± 24.47	294.34 ± 25.07	<0.001
Tp-e ms	91.82 ± 11.82	74.62 ± 10.23	<0.001
Tp-e/QT	0.29 ± 0.27	0.21 ± 0.03	0.006
Tp-e/QTc	0.26 ± 0.26	0.20 ± 0.03	0.033
Tp-e/JT	0.40 ± 0.43	0.27 ± 0.05	0.011
Tp-e/JTc	0.35 ± 0.41	0.26 ± 0.05	0.044

PDM, prediabetes mellitus; bpm, beats per minute; ms, milliseconds; QTc, corrected QT interval; QTd, the difference between the maximum and smallest QT intervals; QTdc, corrected QTd; Tp-e, the distance between the peak and endpoint of the T wave; JTc, corrected JT interval; QT, the distance from the beginning of the Q wave to the end of the T wave; QRS, the distance between Q and S waves; JT, the distance between the T wave’s termination and point J; ECG, electrocardiogram.

**Fig. 3.**
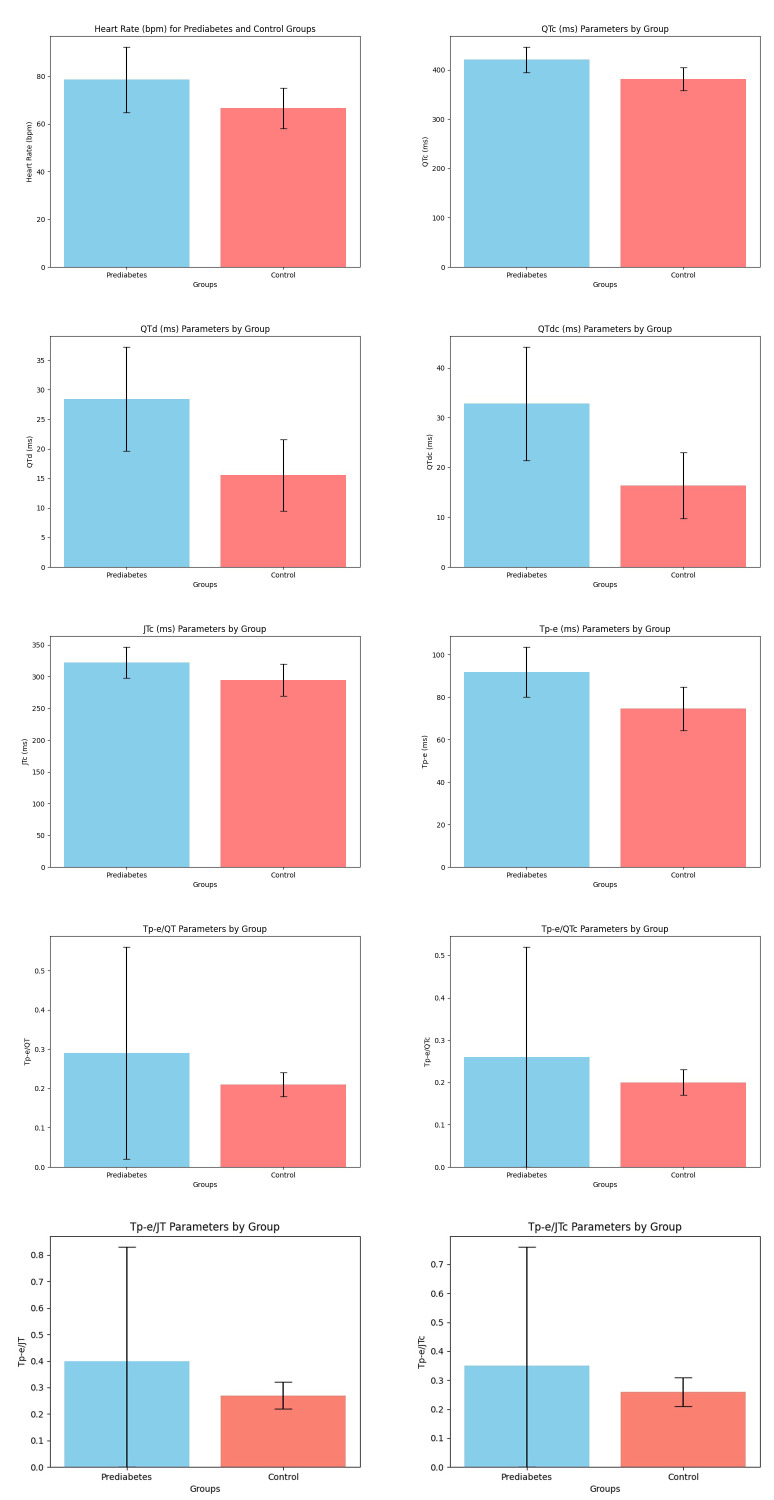
**Comparison of ventricular repolarization parameters on the ECG according to groups**. ECG, electrocardiogram; QTc, corrected QT interval; QTd, the difference between the maximum and smallest QT intervals; QTdc, corrected QTd; JTc, corrected JT interval; Tp-e, the distance between the peak and endpoint of the T wave; QT, the distance from the beginning of the Q wave to the end of the T wave; JT, the distance between the T wave’s termination and point J; bpm, beats per minute.

Fig. 3 compares the ventricular repolarization values on the ECG according to group. The heart rate and Tp-e/QT, Tp-e/QTc, Tp-e/JT, and Tp-e/JTc ratios were statistically substantially greater in the PDM group compared to the control group. Moreover, the QTc, QTd, QTdc, JTc, and Tp-e intervals were statistically longer in the PDM group than in the control group.

The correlation analysis between HbA1c and ventricular repolarization on the ECG is presented in Table 3. The heart rate (r = 0.418, *p* < 0.001), and the QTc (r = 0.582, *p* < 0.001), QTd (r = 0.601, *p* < 0.001), QTdc (r = 0.610, *p* < 0.001), JTc (r = 0.475, *p* < 0.001), and Tp-e (r = 0.592, *p* < 0.001) intervals ​​were found to be statistically significant and there was a moderate positive correlation between these intervals and the HbA1c value. The HbA1c value showed a weak positive correlation with Tp-e/QT (r = 0.178, *p* = 0.028) and Tp-e/JT (r = 0.162, *p* = 0.047) ratios, which were statistically significant.

**Table 3.** **Correlations of HbA1c levels with ECG parameters**.

	HbA1c (%)
Heart rate (bpm)	r = 0.418, *p* < 0.001
QT ms	r = 0.154, *p* = 0.059
QTc ms	r = 0.582, *p* < 0.001
QTd ms	r = 0.601, *p* < 0.001
QTdc ms	r = 0.610, *p* < 0.001
QRS	r = 0.077, *p* = 0.343
JT ms	r = 0.097, *p* = 0.236
JTc ms	r = 0.475, *p* < 0.001
Tp-e ms	r = 0.592, *p* < 0.001
Tp-e/QT	r = 0.178, *p* = 0.028
Tp-e/QTc	r = 0.133, *p* = 0.103
Tp-e/JT	r = 0.162, *p* = 0.047
Tp-e/JTc	r =0.122, *p* = 0.134

HbA1c, hemoglobin A1c; bpm, beats per minute; ms, milliseconds; QTc, corrected QT interval; QTd, the difference between the maximum and smallest QT intervals; QTdc, corrected QTd; Tp-e, the distance between the peak and endpoint of the T wave; JTc, corrected JT interval; QT, the distance from the beginning of the Q wave to the end of the T wave; QRS, the distance between Q and S waves; JT, the distance between the T wave’s termination and point J; ECG, electrocardiogram.

**Fig. 4.**
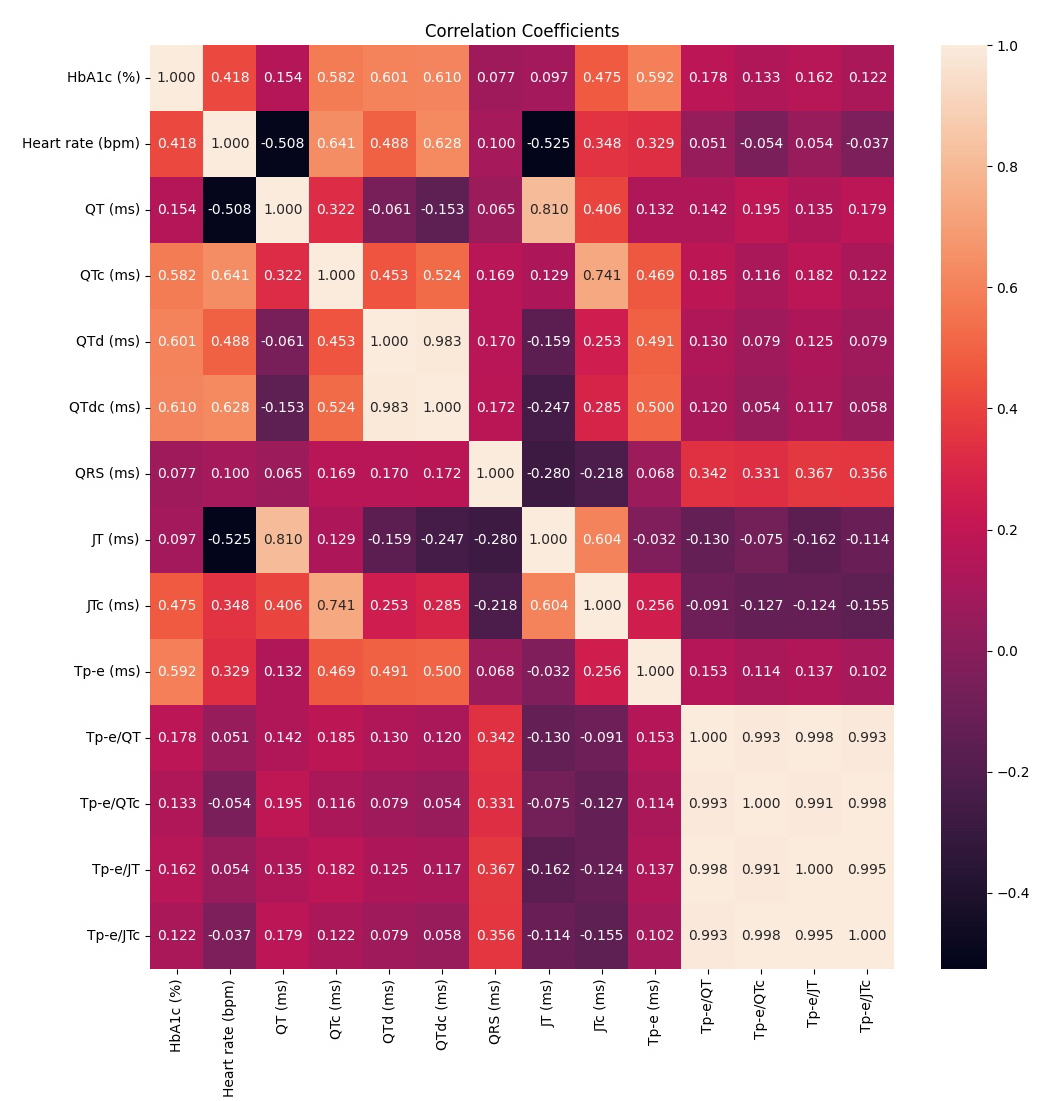
**Heatmap correlation coefficients**. HbA1c, hemoglobin A1c; QT, the distance from the beginning of the Q wave to the end of the T wave; QRS, the distance between Q and S waves; QTc, corrected QT interval; QTd, the difference between the maximum and smallest QT intervals; QTdc, corrected QTd; Tp-e, the distance between the peak and endpoint of the T wave; JTc, corrected JT interval; JT, the distance between the T wave’s termination and point J; bpm, beats per minute; ms, milliseconds.

The heatmap correlation coefficients are presented in Fig. 4. The heart rate (r = 0.418, *p* < 0.001), QTc (r = 0.582, *p* < 0.001), QTd (r = 0.601, *p* < 0.001), QTdc (r = 0.610, *p* < 0.001), JTc (r = 0.475, *p* < 0.001), and Tp-e (r = 0.592, *p* < 0.001) intervals were found to be significant. There was a moderate positive correlation between these intervals and the HbA1c value. Comparatively, the Tp-e/QT (r = 0.178, *p* = 0.028) and Tp-e/JT (r = 0.162, *p* = 0.047) ratios were found with HbA1c value showed a weak positive correlation.

## 4. Discussion

The main finding of our study was that the heart rate (bpm) and QTc, QTd, QTdc, JTc, and Tp-e intervals were significantly higher in PDM patients than in the control group. Furthermore, all ECG parameters correlated with HbA1c levels.

From a social and economic perspective, type 2 DM represents a serious global health issue; meanwhile, PDM constitutes a high-risk factor for developing DM. A meta-analysis has shown that individuals with PDM progress to DM at an annual rate of 3.5–7.0% [12]. Therefore, preventing or managing PDM is essential in reducing the risk of DM onset; moreover, lowering plasma glucose is known to mitigate complications related to DM. Compared to individuals with normoglycemia, those with PDM are at a higher risk of CVD [12, 13].

A recent study examining 3412 individuals aged 71 to 90 assessed the prevalence of PDM and the risk of developing DM. Regardless of the criteria of prediabetes, less than 12% of older persons developed diabetes during the 6.5-year follow-up period. Thus, the progression risk from PDM to DM appears to be lower in older people than in middle-aged individuals [14].

While individuals with PDM experience the same microvascular, macrovascular, and non-vascular complications as those with DM, these occur less frequently. Nonetheless, evidence increasingly supports the positive effects of early intervention in PDM individuals [15]. In a study by Erken Pamukcu *et al*. [16], DM patients with proliferative retinopathy or macro- and microalbuminuria exhibited higher Tp-e/QTc ratios. Another study found that ECG parameters indicating ventricular repolarization, such as the Tp-e interval, Tp-e/QT, and Tp-e/QTc ratios, were elevated in PDM patients. A positive correlation was observed between HbA1c and serum glucose levels and these parameters [17].

In our study, the heart rate and Tp-e/QT, Tp-e/QTc, Tp-e/JT, and Tp-e/JTc ratios in the PDM group were statistically greater than in the control group. Additionally, the QTc, QTd, QTdc, JTc, and Tp-e intervals in the PDM group were statistically longer than those in the control group. Therefore, recognizing and addressing PDM as early DM could improve outcomes since PDM has been shown to increase both mortality and CVD risk, as noted by recent suggestions to reframe the terminology [15, 18]. Hence, new ECG parameters related to ventricular repolarization can help predict mortality and morbidity. In our study, PDM patients demonstrated significantly elevated heart rates and QTc, QTd, QTdc, JTc, and Tp-e intervals compared to controls.

The prevalence of PDM is approximately 20% in adolescents (ages 12–18) and around 25% in young adults (ages 19–34) [19]. PDM individuals are at a higher risk of DM, CVD, kidney disease, and mortality. Lifestyle modification remains the primary intervention for managing PDM. Unlike glucose-based definitions, HbA1c provides several advantages, including its strong association with adverse outcomes. PDM often precedes DM, with both conditions impacting systolic and diastolic heart function and becoming more common with age [20]. A study by Şimşek [21] demonstrated that the hyperglycemic phase in DM patients significantly increased ECG parameters related to the repolarization period, the most vulnerable phase for fatal ventricular arrhythmias.

The QT interval on the ECG reflects the cardiac depolarization and repolarization phases. The longest part of this interval is the repolarization period, the most vulnerable phase for arrhythmias in the myocardium. In diseases such as coronary artery disease (CAD), heart failure, and sudden cardiac death, a longer QT interval is linked to a higher risk of arrhythmias and cardiovascular mortality [22]. The JT interval specifically measures ventricular repolarization, and a study suggests it may be a more accurate marker than the QT interval [23]. For patients with prolonged QRS duration (≥120 ms), the JT interval is recommended for assessing torsades de pointes risk when QT prolongation is observed [24].

Markers of increased ventricular repolarization dispersion include the Tp-e interval, Tp-e/QT, and Tp-e/QTc ratios. A prolonged Tp-e interval on the ECG due to irregularities in transmural repolarization is also linked to a heightened risk of polymorphic ventricular tachycardia [25]. In a study by Gürler and İnanır [26], DM patients with CAD had significantly higher repolarization markers than those with normal coronary arteries.

In our investigation, the heart rate and Tp-e/QT, Tp-e/QTc, Tp-e/JT, and Tp-e/JTc ratios were statistically significantly higher in the PDM group than in the control group. The QTc, QTd, QTdc, JTc, and Tp-e intervals of the PDM group were likewise statistically significantly longer than those in the control group. An ECG is an accessible and valuable tool for assessing arrhythmia risk. Our study has demonstrated alterations in repolarization parameters among PDM patients, and these changes, suggest that patients may be predisposed to arrhythmias. However, the processes through which ventricular repolarization markers can be used in clinical settings to classify risk in PDM patients remain debatable. Thus, more randomized controlled trials are needed before comprehensive recommendations can be made.

## 5. Limitations

Two major drawbacks in this study are the small sample size and the manual measurement computation. Although QT measurement has improved thanks to automated analytic methods, problems remain in this procedure. Electrode cable variability makes it harder to detect the T wave endpoint (T-end) reliably in ECGs. Automated techniques might be preferred because a manual T-end study is not reproducible [27, 28]. Another limitation is that we did not evaluate how medication and lifestyle modifications (diet, exercise) affected the repolarization parameters of the participants in the control group.

## 6. Conclusions

In our study, the averages for age, gender, smoking, hyperlipidemia, and BMI were similar for the two groups. Likewise, the QT, QRS, and JT intervals were comparable in both groups. In comparison to the control group, PDM patients had significantly higher heart rates and QTc, QTd, QTdc, JTc, and Tp-e intervals. The findings of this study demonstrated that the HbA1c value exhibited a moderate positive correlation with heart rate as well as with QTc, QTd, QTdc, JTc, and Tp-e intervals. All of these associations were found to be statistically significant, indicating a meaningful relationship between HbA1c levels and these ECG parameters. Furthermore, the analysis revealed that the HbA1c value had a weak but still significant positive correlation with the Tp-e/QT and Tp-e/JT ratios, further emphasizing its relevance in this context. Changes in ECG parameters in these patients suggest the possibility that they may be at risk for arrhythmias.Our research has shown that PDM patients have changes in their repolarization parameters and showed that patients with PDM are prone to ventricular arrhythmia in the early period of the disorder. However, more randomized clinical studies are needed on this subject.

## Availability of Data and Materials

The datasets used and/or analyzed during the current study are available from the corresponding author on reasonable request.
